# Management of Body Condition Score between Pregnancy Scanning and Lamb Marking Impacts the Survival of Triplet-Bearing Ewes and Their Lambs

**DOI:** 10.3390/ani13132057

**Published:** 2023-06-22

**Authors:** Emmanuelle Haslin, Travis Allington, Sarah E. Blumer, Johan Boshoff, Bronwyn E. Clarke, Serina N. Hancock, Gavin A. Kearney, Paul R. Kenyon, Jarryd Krog, Lyndon J. Kubeil, Amy Lockwood, Gordon Refshauge, Jason P. Trompf, Andrew N. Thompson

**Affiliations:** 1 School of Agriculture and Environment, Massey University, Tennent Drive, Palmerston North 4410, New Zealand; 2 Centre for Animal Production and Health, Murdoch University, 90 South Street, Murdoch, WA 6150, Australia; 3 Faculty of Science, Agriculture, Business and Law, University of New England, Elm Avenue, Armidale, NSW 2351, Australia; 4 36 Paynes Road, Hamilton, VIC 3300, Australia; 5 Department of Economic Development, Jobs, Transport and Resources, 89 Sydney Road, Benalla, VIC 3672, Australia; 6 New South Wales Department of Primary Industries, Cowra Agricultural Research and Advisory Station, 296 Binni Creek Road, Cowra, NSW 2351, Australia; 7 J.T. Agri-Source, 2A Bradley Drive, Melbourne, VIC 3082, Australia

**Keywords:** triplet, ewe mortality, lamb survival, body condition score, Merino ewes, Maternal ewes

## Abstract

**Simple Summary:**

Body condition score (BCS) is an assessment of the amount of fat and muscle covering the lumbar spine and short ribs of sheep. It is widely used as a management tool to assess the nutritional status of sheep. This study investigated whether managing triplet-bearing ewes at a higher or lower BCS between pregnancy scanning and lamb marking impacted the survival of the ewes or their lambs. Triplet-bearing ewes of Maternal (crossbred or composite) or Merino breed were allocated to one of two treatments at pregnancy scanning: ‘High’ or ‘Low’ BCS. The BCS of individual ewes was assessed at pregnancy scanning, pre-lambing and marking, and ewe and lamb mortality to marking, recorded for each mob. Survival of triplet-bearing Merino ewes and their lambs was greater when ewes were managed at the High BCS compared to the Low BCS. The BCS treatment had no effect on the survival of Maternal ewes or their lambs. Survival of triplet-born Merino but not Maternal lambs was greater when ewes had a greater BCS pre-lambing or gained BCS between pregnancy scanning and pre-lambing. Triplet-bearing ewes of Maternal and Merino breed that gained BCS between pregnancy scanning and pre-lambing had greater survival to marking. Producers should, therefore, manage the nutrition of triplet-bearing Merino ewes so that ewes are in greater BCS at lambing and/or to gain BCS between pregnancy scanning and lambing to improve ewe and lamb survival. Triplet-bearing Maternal ewes should be managed to gain BCS between pregnancy scanning and lambing to improve ewe survival.

**Abstract:**

This study evaluated the impacts of management of body condition score (BCS) between pregnancy scanning and lamb marking on the mortality of triplet-bearing ewes and their lambs at 19 research sites across Southern Australia. Triplet-bearing ewes of Maternal (crossbred or composite) or Merino breed were randomly allocated to treatment at pregnancy scanning at an average of 97 days from the start of joining: High or Low BCS. The BCS of individual ewes was assessed at pregnancy scanning, pre-lambing (average of 137 days from the start of joining) and marking (average of 165 days from the end of joining), and ewe and lamb mortality to marking, recorded for each mob. The average BCS at pregnancy scanning was 3.4 for Maternal ewes and 3.3 for Merino ewes. There were no breed by BCS treatment effects on the BCS of ewes at pregnancy scanning or lamb marking or on the change in BCS between pregnancy scanning and pre-lambing or between pre-lambing and marking. The change in BCS differed between the High and Low BCS treatments, between pregnancy scanning and pre-lambing (0.12 vs. −0.33; *p* < 0.001) and between pre-lambing and marking (−0.39 vs. 0.07; *p* < 0.001) but did not differ between breeds. The average BCS at marking for ewes managed at the High and Low BCS treatments was 3.1 and 3.0 for Maternals and 3.0 and 2.8 for Merinos. Survival of triplet-bearing Merino ewes (*p* < 0.01) and their lambs (*p* < 0.001) was greater when ewes were managed at the High BCS compared to the Low BCS. The BCS treatment did not impact the survival of Maternal ewes or their lambs. The survival of Merino but not Maternal lambs was higher when ewes were in greater BCS pre-lambing (*p* < 0.01) and when ewes gained BCS between pregnancy scanning and pre-lambing (*p* < 0.01). Ewe mortality was lower when ewes gained BCS between pregnancy scanning and pre-lambing (*p* < 0.05). Merino ewes were more likely to die than Maternal ewes for a given change in BCS between pregnancy scanning and pre-lambing (*p* = 0.065). Overall, our findings demonstrate that producers should manage the nutrition of triplet-bearing Merino ewes so that ewes are in greater BCS at lambing and/or to gain BCS between pregnancy scanning and lambing to improve ewe and lamb survival. Triplet-bearing Maternal ewes should be managed to gain BCS between pregnancy scanning and lambing to improve ewe survival.

## 1. Introduction

Increasing the number of lambs weaned per ewe has a positive impact on the profitability of sheep enterprises [1,2,3]. The number of lambs weaned per ewe joined under pastoral systems, such as in Australia and New Zealand, has risen by 10 to 20% over the last twenty years [4,5]. However, a consequence of greater fecundity is an increase in the number of triplet-bearing ewes within a flock [6,7]. Triplet-born lambs are smaller at birth, can be metabolically challenged, and receive less colostrum and milk than their twin counterparts, contributing to lower survival to weaning [8]. In addition, although data are somewhat sparse, triplet-bearing ewes themselves have lower survival during late-pregnancy and in lactation compared to single- and twin-bearing ewes [7,9], and this lower survival has a negative impact on lamb survival [5,7]. Australian producers who scanned their ewes for triplets were surveyed in 2018 and reported that the average mortality of triplet-bearing ewes between pregnancy scanning and marking was 6.4% and ranged from 0 to 27%, regardless of breed. However, the reported survival of triplet-born lambs was significantly higher for Maternals, comprising composite and crossbred breeds, than Merinos (60.1% vs. 52.9%) [7]. Therefore, developing management guidelines for triplet-bearing ewes to increase survival of the ewes and their lambs will improve productivity and animal welfare.

The considerable nutritional demand of triplet-bearing ewes in late pregnancy and lactation is often not matched by feed intake, especially under pastoral conditions [8], and is a likely contributor to mortality of triplet-bearing ewes and their lambs. Ewes rely on their whole-body energy reserves to meet the deficit when nutritional needs are not met [10]. Body condition score (BCS), which is a subjective measure of whole-body energy reserves [11], is a well-established tool for assessing the nutritional status of sheep and enables them to be managed to meet BCS targets [10]. Positive relationships have been identified between BCS in mid- and late-pregnancy and ewe milk production [12,13], lamb birthweight [14,15], and lamb survival [14,16,17]. A positive relationship was also reported between BCS pre-joining and ewe survival [18]. Therefore, it might be expected that the survival of triplet-bearing ewes and their lambs to weaning would be improved by ewes being in higher BCS pre-lambing or at least maintaining BCS during late pregnancy. However, there appears to be a paucity of data available examining the impacts of BCS and BCS change in pregnancy on the performance of triplet-bearing ewes, especially at a commercial scale. McCoard et al. [19] evaluated the impacts of BCS on lamb survival when ewes grazed turnip and swede crops during mid-gestation in New Zealand. The authors reported that the survival of triplet-born lambs decreased from 72–73% to 57–58% when triplet-bearing ewes lost 1 BCS between pregnancy scanning and approximately 120 days of gestation. The limited pastoral-based studies that have been undertaken in New Zealand using maternal breeds found no effect of BCS in mid- to late-pregnancy on lamb birthweight and survival, although these studies were limited by the small range in BCS [20,21,22]. More recently, studies in Australia have reported that greater liveweight of triplet-bearing ewes in late-pregnancy increased the birthweight and survival of their lambs [23,24]. A higher BCS of Maternal ewes in late pregnancy has also been reported to increase the birthweight and survival of their multiple-born lambs [24,25].

A recent survey of sheep producers in Australia reported that many producers ranked BCS at lambing as the management practice of highest priority for further research to understand the impacts on the survival of triplet-bearing ewes and their lambs [7]. There was no apparent difference in the survival of triplet-bearing ewes and their lambs between farms that did or did not rank BCS as a high priority. However, there were differing opinions in what producers considered to be an optimal BCS for triplet-bearing ewes at lambing, with a range between 2.8 and 3.5. This is similar to Kenyon et al. [10], who suggested that the optimal BCS is likely in a range of 3.0 to 3.5. Therefore, the aim of this study was to evaluate the impacts of BCS management between pregnancy scanning and marking on the survival of Maternal and Merino triplet-bearing ewes and their lambs under commercial conditions across Australia. It was hypothesised that the survival of triplet lambs born to ewes managed at the High BCS would be greater than those managed at the Low BCS, regardless of ewe breed.

## 2. Materials and Methods

### 2.1. Sites and Experimental Design

Research sites were established on 19 commercial farms across New South Wales (NSW; *n* = 8), South Australia (SA; *n* = 3), Victoria (Vic; *n* = 4), and Western Australian (WA; *n* = 4) between 2019 and 2021. Each site involved either mixed-aged, triplet-bearing Maternal (*n* = 12 sites) or Merino (*n* = 7 sites) ewes. Maternal refers to crossbred or composite sheep breeds used for production of prime lambs. One farm with Merinos was used in both 2020 and 2021, and these are counted as separate research sites. The total number of mobs and ewes per treatment is summarised in Table 1. 

The average length of joining across the research sites was 36 days. After pregnancy scanning, at an average of 97 days from the start of joining, ewes at each research site were randomly allocated to one of two treatments: ‘High’ or ‘Low’ BCS. Treatments at each site were replicated where adequate ewes and lambing paddocks were available. On average, each Maternal site included two replicates per treatment (range of 1–4 replicates/treatment/site) and each Merino site included three replicates per treatment (range of 1–5 replicates/treatment/site). The aim was for the average BCS for treatments within each research site to differ by at least 0.3 BCS units by the pre-lambing assessment. The target BCS for each treatment was determined based on the average BCS of triplet-bearing ewes at allocation to treatments. The BCS differences were achieved by either allocating the treatments to paddocks with differing Feed-On-Offer (FOO) and/or altering the rates of supplementary feeding.

### 2.2. Animal and Paddock Measurements

The BCS of all ewes was assessed when ewes were allocated to treatments at pregnancy scanning. All ewes were then re-assessed at approximately 110 days from the start of joining to determine whether management needed to be adjusted to ensure the pre-lambing BCS targets were met. The BCS of all ewes was then assessed at pre-lambing (average of 137 days from the start of joining) before ewes were allocated to their lambing paddocks and finally at lamb marking (average of 165 days from the end of joining). Ewe BCS was assessed by a single operator at each research site, as described by Jefferies [26], using 0.2 to 0.3 score increments.

Lambing paddocks between treatments and replicates within sites were selected to be as similar as possible for characteristics including FOO, pasture composition, the amount and type of shelter, paddock size and therefore ewe stocking rate. The characteristics of each lambing paddock were visually assessed by a single assessor at each research site. Paddock topography and shelter availability were characterised as described by Lockwood et al. [27]. 

At lamb marking, ewes had their udder manually palpated to identify whether they were lactating or not, i.e., ‘dry’, and the number of live ewes and lambs per mob were counted. Lamb survival was calculated for each mob based on the number of foetuses identified to ewes present pre-lambing and the number of live lambs at marking. Ewe deaths were determined via identification of dead ewes between pregnancy scanning and lamb marking. Ewes that were allocated to treatments at pregnancy scanning but were absent at the pre-lambing and/or marking measurements were also classified as dead. Ewe mortality during the lambing period at the paddock level was calculated based on the number of ewes allocated to lambing paddocks and the number of ewes present at lamb marking.

### 2.3. Pasture Measurements

Feed-On-Offer was visually assessed (kg dry matter (DM)/ha) at 25 locations in each of the lambing paddocks pre-lambing and at lamb marking at each research site. The percentage of legume in the pasture was also assessed at the same sites. The visual assessments of FOO were calibrated against ten 0.1 m^2^ quadrat cuts taken from each lambing paddock. Pasture within each quadrat was harvested to ground level, and samples were dried and then weighed to determine the DM content. The average FOO and proportion of legumes in the paddocks for each treatment pre-lambing and at marking are provided in Table 2.

### 2.4. Weather Conditions during Lambing

Daily data for temperature, rainfall and windspeed between day 145 from the start of joining and marking were collected via the Australian Gridded Climate Data (AGCD) and Australian Community Climate and Earth-System Simulator (ACESS-G) services from the Bureau of Meteorology for each research site. Windspeed at 10 m was provided by the Bureau of Meteorology and was converted to lamb height of 0.4 m using the formula described by Thornley and Johnson [28]. Daily chill index was calculated for each research site using the formula described by Nixon-Smith [29], with weighting of daily temperature (0.75 × maximum temperature + 0.25 × minimum temperature), as per Horton et al. [30]. The average chill index between day 145 from the start of joining and marking was then calculated for each research site. The average chill index across all research sites was 733 kJ/m^2^/h, with a range in averages from 639 to 799 kJ/m^2^/h.

### 2.5. Statistical Analysis

Data were analysed via the following methods using GENSTAT 22nd edition (VSN International 2022, Hemel Hempstead, UK) and SAS v9.4 (SAS Institute Inc., Cary, NC, USA). 

Ewe BCS at pregnancy scanning, pre-lambing, and marking, and the BCS change between pregnancy scanning and pre-lambing, and pre-lambing and marking were analysed separately at the paddock level using the method of restricted maximum likelihood (REML). Breed (Maternal vs. Merino), BCS treatment (High vs. Low), and their two-way interaction was fitted as fixed effects and year, site nested within year, and paddock (i.e., mob) nested within site, all included as random effects.

Lamb survival and ewe mortality at the paddock level were analysed using REML with the BCS treatment, breed, proportion of shelter available in the lambing paddocks, the average chill index between pre-lambing and marking, and interactions thereof, where appropriate, fitted as fixed effects. Year, site nested with year, and paddock nested within site were fitted as random effects. Once BCS treatment and breed effects were determined, the lamb survival model was re-run with the average BCS of ewes pre-lambing instead of BCS treatment as a fixed effect.

Estimates of individual ewe mortality and the probability of an individual ewe to be lactating at marking were assessed through fitting Generalized Linear Mixed Models (GLMMs). The approach used a logit transformation and binomial distribution. The probability of ewes to be lactating at marking included either alive ewes at marking only (i.e., lactating and non-lactating ewes) or alive and dead ewes at marking, where dead ewes were considered as non-lactating. Using additive models, logits were predicted as a function of breed, BCS at pregnancy scanning, and change in BCS from pregnancy scanning to pre-lambing as fixed effects. Year and site nested within year were fitted as random effects. Both BCS variates were tested for quadratic effects. All possible models were examined with statistical significance of terms and interactions thereof accepted at *p* < 0.05.

## 3. Results

### 3.1. Ewe Body Condition Score

The average BCS at pregnancy scanning was 3.4 for Maternal ewes and 3.3 for Merino ewes (l.s.d. = 0.36). There was no breed by BCS treatment effect on the BCS of ewes at pregnancy scanning (*p* = 0.221). There was also no breed by BCS treatment effect on the change in BCS between pregnancy scanning and pre-lambing or between pre-lambing and marking (Table 3). The change in BCS differed between the High and Low BCS treatments, between pregnancy scanning and pre-lambing (0.12 vs. −0.33; *p* < 0.001) and between pre-lambing and marking (−0.39 vs. 0.07; *p* < 0.001), but did not differ between breeds. The average BCS at marking for ewes managed at the High and Low BCS treatments was 3.1 and 3.0 for Maternals and 3.0 and 2.8 for Merinos (l.s.d. within breed = 0.09; l.s.d. between breeds = 0.19). There was no breed by BCS treatment effect on the BCS of ewes at marking (*p* = 0.244).

### 3.2. Effects of Treatment and Ewe Breed on Lamb Survival to Marking

There was an effect of breed by BCS treatment on lamb survival to marking, where triplet lambs born to Merino ewes in the High BCS treatment had greater survival to marking than their counterparts born to ewes in the Low BCS treatment (Table 4). Body condition score treatment had no effect on the survival of lambs born to Maternal ewes. Lamb survival to marking did not differ between Maternal and Merino ewes within the High BCS treatment. However, Merino lambs born to ewes in the Low BCS treatment had lower survival than their Maternal counterparts (Table 4). The proportion of shelter available in the lambing paddocks and the average chill index during lambing had no effect on the survival of triplet-born lambs, nor were there any interactions with ewe BCS treatment.

### 3.3. Effects of BCS at Lambing and BCS Change between Pregnancy Scanning and Pre-Lambing on Lamb Survival to Marking

**Figure 1:**
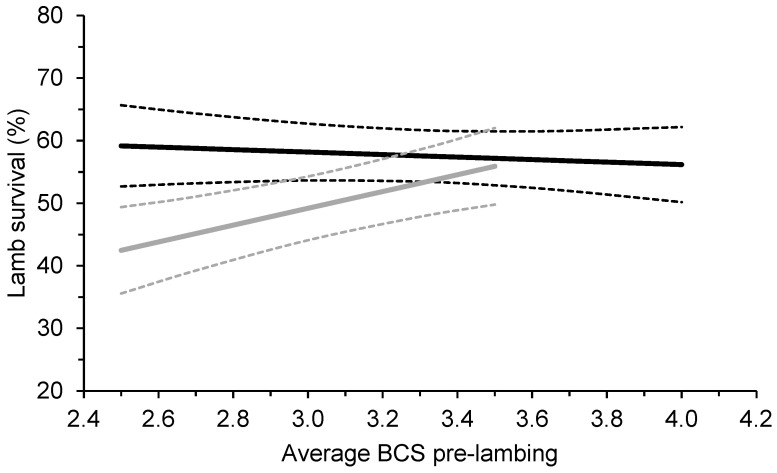
Effect (±95% confidence intervals; dotted lines) of the average body condition score (BCS) of mobs of triplet-bearing Maternal (black lines) and Merino (grey lines lines) ewes pre-lambing (average of 136 days from the start of joining) on the survival of their lambs to marking at 19 commercial research sites across southern Australia between 2019 and 2021. The average BCS at pregnancy scanning was 3.4 for Maternals and 3.3 for Merinos.

There was a significant effect of breed by the average BCS of ewes at lambing on lamb survival, where the survival of Merino but not Maternal lambs was higher when ewes were in greater BCS at lambing (*p* < 0.01; Figure 1). On average, the survival of Merino lambs to marking was 6.7% higher when the average pre-lambing BCS of mobs of triplet bearing was 0.5 BCS greater, within a range of BCS 2.5 to 3.5 (Figure 1).

**Figure 2:**
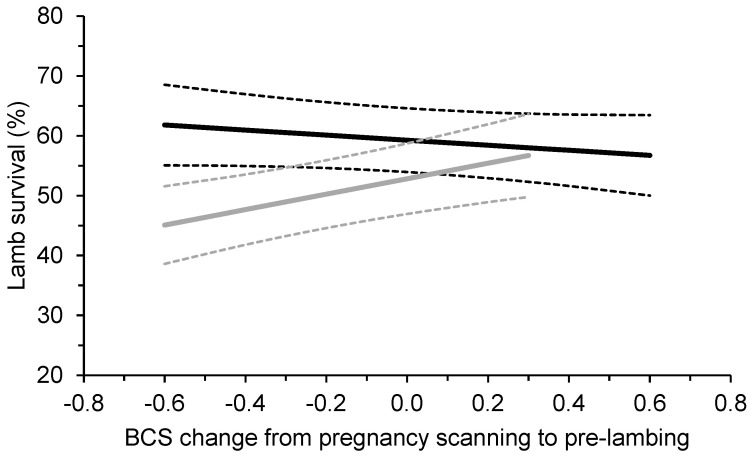
Effect (±95% confidence intervals; dotted lines) of the average change in body condition score (BCS) of mobs of triplet-bearing Maternal (black lines) and Merino (grey lines) ewes between pregnancy scanning and pre-lambing on the survival of their lambs to marking at 19 commercial research sites across Southern Australia between 2019 and 2021. On average, measurements for pregnancy scanning were conducted at 97 days from the start of joining and pre-lambing were conducted at 136 days from the start of joining. The average BCS at pregnancy scanning was 3.4 for Maternals and 3.3 for Merinos.

There was a significant effect of breed by the average BCS change in ewes between pregnancy scanning and pre-lambing on lamb survival, where gaining BCS increased the survival of Merino lambs but not Maternals (*p* < 0.01; Figure 2). On average, the survival of Merino lambs to marking increased by 6.5% when ewes gained 0.5 BCS between pregnancy scanning and pre-lambing (Figure 2).

### 3.4. Effects of Treatment and Ewe Breed on Ewe Mortality to Marking

There was an effect of breed by BCS treatment on ewe mortality, where Merino ewes in the Low BCS treatment had greater mortality to marking than their counterparts in the High BCS treatment (Table 4). By contrast, BCS treatment had no effect on the mortality of Maternal ewes. Ewe mortality to marking did not differ between Maternal and Merino ewes within the High BCS treatment. However, Merino ewes managed at a Low BCS had greater mortality than their Maternal counterparts (Table 4).

### 3.5. Effect of BCS Change between Pregnancy Scanning and Pre-Lambing on Ewe Mortality to Marking

**Figure 3:**
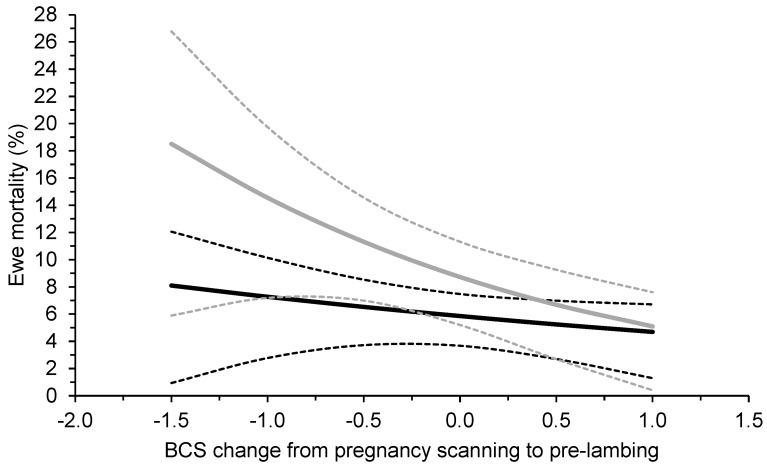
The effect (±95% confidence intervals; dotted lines) of the change in body condition score (BCS) of triplet-bearing Maternal (black lines) and Merino (grey lines) ewes between pregnancy scanning and pre-lambing on their mortality to marking at 19 commercial research sites across southern Australia between 2019 and 2021. On average, measurements for pregnancy scanning were conducted at 97 days from the start of joining and those pre-lambing were conducted at 136 days from the start of joining. The average BCS at pregnancy scanning was 3.4 for Maternals and 3.3 for Merinos.

Ewe BCS at pregnancy scanning had no effect on ewe mortality. There was no effect of breed by BCS change between pregnancy scanning and pre-lambing on ewe mortality (*p* = 0.290; Figure 3). Ewe mortality decreased as the BCS change between pregnancy scanning and pre-lambing increased (*p* < 0.05). Merino ewes were more likely to die than Maternal ewes for a given change in BCS between pregnancy scanning and pre-lambing (*p* = 0.065).

### 3.6. Effect of BCS Change between Pregnancy Scanning and Pre-Lambing on the Probability of the Ewe Lactating at Marking

**Figure 4:**
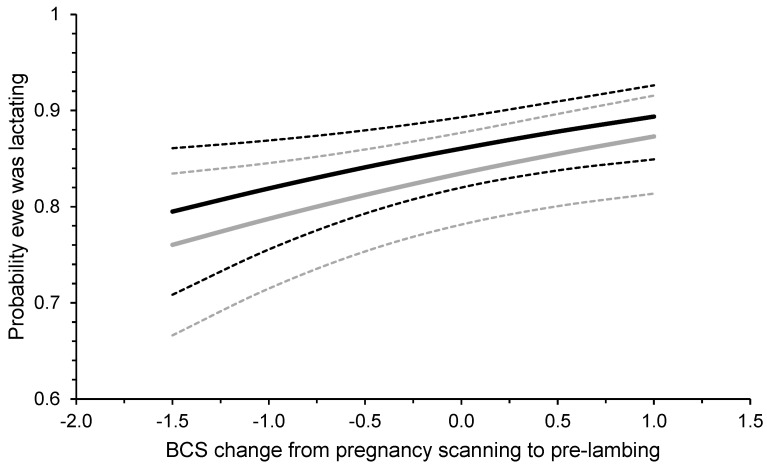
Prediction (±95% confidence intervals; dashed lines) of the relationship between the change in ewe body condition score (BCS) between pregnancy scanning and pre-lambing and the probability of triplet-bearing Maternal (black lines) and Merino (grey lines) ewes to be lactating at marking at 19 commercial research sites across southern Australia between 2019 and 2021. Ewes whose entire litter died and ewes that died prior to marking were included in the analysis. On average, measurements for pregnancy scanning were conducted at 97 days from the start of joining and those pre-lambing were conducted at 136 days from the start of joining. The average (±SD) BCS of individual ewes at pregnancy scanning was 3.4 ± 0.56 for Maternals and 3.3 ± 0.43 for Merinos for the raw data.

Ewe BCS at pregnancy scanning, the change in BCS between pregnancy scanning and pre-lambing, and ewe breed had no effect on the probability of those ewes alive at marking being non-lactating. When ewes that died before marking were also considered as ‘non-lactating’ at marking, BCS at pregnancy scanning and breed remained non-significant. However, there was a positive relationship between BCS change between pregnancy scanning and pre-lambing on the probability for a triplet-bearing ewe to be lactating at marking (*p* < 0.01; Figure 4).

## 4. Discussion

The survival of triplet lambs born to Maternal ewes did not differ between BCS treatments. However, lambs born to Merino ewes in the High BCS treatment had greater survival than those born to ewes in the Low BCS treatment. In addition, the survival of lambs born to triplet-bearing Merino ewes was greater when the average BCS of ewes pre-lambing and when ewes gained BCS between pregnancy scanning and lambing. This was not observed for their Maternal counterparts. Therefore, our hypothesis was partially supported. 

The average BCS of the Merino ewes was 3.3 at pregnancy scanning. Merino ewes managed at a High BCS maintained BCS between pregnancy scanning and pre-lambing whilst those managed at a Low BCS lost 0.4 BCS. Both treatments lost BCS between pre-lambing and marking, although those ewes managed at the High BCS had greater BCS loss. Late pregnancy represents the greatest period of foetal growth [31,32] and hence nutrition during this period dictates lamb birthweight [33]. It is well known that lambs born to Merino ewes in poorer BCS during late-pregnancy or that lose liveweight during late pregnancy have lower birthweights and thus poorer survival [15,23]. Our findings also showed that the survival of lambs born to triplet-bearing Merino ewes was poorer when ewes had a lower BCS pre-lambing or lost BCS between pregnancy scanning and pre-lambing. Hence, the poorer survival of triplet lambs born to Merino ewes managed at a Low BCS can be explained by the loss in ewe BCS between pregnancy scanning and lambing and the overall lower BCS of these ewes compared with those managed at the High BCS. It has been reported that triplet-bearing ewes have similar feed intakes to twin-bearing ewes under pastoral conditions [34], despite them having greater nutritional demands and hence triplet-bearing ewes have been reported to be in negative energy balance during late pregnancy [8]. Ewes in poorer condition have less ability to buffer any nutritional shortfalls through mobilising their body reserves and are subsequently at greater risk of metabolic disease, dystocia and death [35]. The greater mortality of triplet-bearing Merino ewes managed at a Low BCS compared with a High BCS can therefore also be explained by the overall poorer BCS and loss in BCS during late pregnancy, predisposing metabolic disease and dystocia. 

The average BCS of the Maternal ewes at pregnancy scanning was 3.4, which was similar to that of the Merino ewes. Maternal ewes managed at a High BCS gained 0.26 BCS between pregnancy scanning and pre-lambing whilst those managed at a Low BCS lost 0.25 BCS. Maternal ewes managed at a High BCS lost more BCS between pre-lambing and marking, resulting in a similar average BCS for the High and Low BCS treatments at marking. BCS treatment had no significant impact on the survival of triplet-born Maternal lambs. These findings suggest that Maternal ewes in BCS 3.4 at pregnancy scanning which lose up to 0.25 BCS, on average, to pre-lambing can compensate for the declining nutritional status, resulting in no negative impact on the survival of their lambs. This may also suggest that a BCS of 3.1–3.2 is within or near the optimal range for triplet-bearing Maternal ewes at lambing and thus their lambs may have been born within the optimal range for birthweight. This is supported by Behrendt et al. [24] who found that undernutrition only decreased lamb birthweight when the BCS of Maternal composite ewes at lambing was less than 3 and that lamb survival was near maximum when ewes were managed to BCS 3.2 to 3.5 at lambing. Similarly, Kenyon et al. [20] observed no effect of the BCS of triplet-bearing Romney ewes on the survival of their lambs when the average BCS of ewes ranged from 2.8 to 3.4 in late pregnancy. However, Kenyon et al. [22] found that the survival of triplet lambs born to ewes that were managed to BCS 2.5 during pregnancy (BCS 2.5 at lambing) was poorer than those born to ewes managed to BCS 3 during pregnancy (BCS 2.7 at lambing). On average, the BCS of Maternal ewes was greater in our study and therefore further exploration of the impacts of lower BCS profiles on ewe and lamb survival is warranted. Our findings also suggest that allowing Maternal ewes to lose significant BCS between pre-lambing and marking could compromise ewe and lamb survival and hence further work is required to investigate this relationship. 

The impact of BCS treatment on lamb survival differed between Merinos and Maternals in our study. Survival was poorer for lambs born to Merino ewes managed at a Low BCS, who lost 0.4 BCS in late pregnancy, compared to those managed at a High BCS, which maintained BCS, whereas lamb survival was not compromised when Maternal ewes managed at a Low BCS lost 0.25 BCS during late pregnancy compared with those managed at a High BCS which gained 0.26 BCS. Furthermore, our results show that lambs born to triplet-bearing Merino ewes that are in greater BCS pre-lambing or which gain BCS between pregnancy scanning and lambing have better survival to marking, whereas this was not observed for Maternals. This contrasts the findings of Hocking-Edwards et al. [25], who suggested that similar coefficients for liveweight change in late pregnancy predict the birthweight and survival of single- and multiple-born crossbred and Merino lambs, noting that most multiple-bearing ewes in this study were twin-bearing ewes with few triplets. 

In addition to the treatment effects, analysis of data from individual ewes showed that there was a negative relationship between the change in BCS between pregnancy scanning and pre-lambing and mortality of Maternal and Merino ewes. Mortality of Merino ewes was more sensitive to BCS change compared with Maternals, with the mortality of Merino ewes increasing considerably when ewes lost more than 0.5 BCS between pregnancy scanning and pre-lambing. Given that ewe deaths cause lamb deaths, this would indicate that triplet-bearing ewes should be managed to ensure that they do not lose more than 0.5 BCS during late pregnancy. Overall, the paddock- and individual-level analyses from our study demonstrate that producers should manage the nutrition of triplet-bearing Merino ewes so that ewes are in greater BCS at lambing and/or to gain BCS between pregnancy scanning and lambing to improve ewe and lamb survival. Triplet-bearing Maternal ewes should be managed to gain BCS between pregnancy scanning and lambing to improve ewe survival.

This study appears to be the first specifically designed to examine the effects of BCS between pregnancy scanning and marking on the survival of triplet-bearing ewes. Ewe mortality between late pregnancy and marking in this study ranged from 5% to 11%. This is similar to or greater than that reported on commercial farms across Australia by Thompson et al. [7] for non-Merino (4.9%) and Merino (6.7%) ewes between pregnancy scanning and marking and by McQuillan et al. [36] for non-Merino ewes (5%) between pre-lambing and marking. However, in contrast to our findings, Capdevila-Ospina et al. [37] reported that the BCS of Coopworth-cross composite ewes between the start of breeding and weaning had no effect on ewe mortality during the lambing period. Ewe mortality included all litter sizes (i.e., single-, twin-, and triplet-bearing ewes) and the data did not differentiate between them, which may explain why these findings are inconsistent with those from our study. A BCS of 2.0 or less pre-lambing has been reported to increase the risk of pregnancy toxemia for ewes, regardless of litter size [38]. Triplet-bearing ewes in the same study were also more likely to suffer from pregnancy toxemia [38]. It is therefore important that further research into the effects of ewe BCS on ewe and lamb survival involves autopsy of dead ewes to determine the association between ewe BCS, timepoint of death and cause of death. This will enable the development of targeted management guidelines and treatment interventions to prevent the death of triplet-bearing ewes and subsequently their lambs. 

It should also be noted that, on average, ewe BCS did not exceed 3.7 for Maternals and 3.3 for Merinos in this study and therefore ewes would not be considered over-fat. Consultation with Australian sheep producers who identified triplet-bearing ewes at pregnancy scanning found that 80% of producers managed ewe BCS to reduce ewe mortality and about 50% of these producers, most of which managed Maternal ewes, indicated that they did so to avoid ewes becoming over-fat [7]. Fatter ewes accumulate fat around the rumen which limits feed intake and subsequently predisposes pregnancy toxemia when energy requirements are not being met, which can lead to ewe death [39]. Fatter ewes and their lambs are predisposed to dystocia [24,40], and pregnancy toxemia also increases the risk of dystocia [35]. Multiple-born lambs are more likely to be malpresented [35] and overstretching of the myometrium can cause uterine inertia in multiple-bearing ewes [41]. Hence, the impacts of being fatter and multiple-bearing are likely to compound the risk of dystocia for fatter triplet-bearing ewes. Therefore, further work is required to determine the impacts of over-conditioning triplet-bearing ewes, particularly those of Maternal breed, on ewe and lamb survival to inform best-practice management guidelines.

Udder assessment was undertaken at marking as a proxy measure of ewes lactating (i.e., rearing of a least one lamb) or not (i.e., all lambs presumed to have died). The proportion of triplet-bearing ewes lactating at marking, when only ewes alive at marking were considered, was not influenced by BCS at pregnancy scanning, BCS change between pregnancy scanning, and pre-lambing or breed. This is consistent with the findings of Griffiths et al. [42]. This result also indicates that the poorer survival of lambs born to Merino ewes in the Low BCS treatment was not associated with increased numbers of ewes losing their entire litter. However, the impact of BCS change between pregnancy scanning and pre-lambing was significant when the individual ewes that died before marking were included in the analysis. This indicates that ewe mortality was a driver of the positive relationship between BCS change and the proportion of ewes lactating at marking. An increase in BCS change decreased individual ewe mortality, increasing the proportion of ewes rearing lambs, and could be related to an increase in the survival of triplet-born lambs. Ewe death in late pregnancy and lactation is likely a significant contributor to overall lamb mortality [5], with a reported correlation of −0.63 between triplet-bearing ewe mortality and the survival of their lambs [7]. In one-year-old ewe lambs, it has recently been reported that the 2.5% of young ewes that died during the lambing period accounted for 11% of total lamb mortalities [43]. In the present study, the estimation of the proportion of lamb deaths related to the death of their dam was, on average, 17.5% of the total lamb mortality but ranged from 0% to 69%. These proportions highlight that reducing the mortality of triplet-bearing ewes by gaining BCS between pregnancy scanning and lambing can be used by producers to improve survival of triplet-born lambs.

## 5. Conclusions

The survival of triplet-bearing Merino ewes and their lambs was greater when ewes were managed at a higher BCS between pregnancy scanning and marking. In contrast, the survival of triplet-bearing Maternal ewes and their lambs was not significantly influenced by the BCS treatments. Losing BCS between pregnancy scanning and pre-lambing decreased the survival of Maternal and Merino ewes, although Merino ewes were more sensitive to BCS change when ewes lost more than 0.5 BCS. Lambs born to triplet-bearing Merino ewes in greater BCS pre-lambing or which gained BCS between pregnancy scanning and lambing had better survival to marking, but this was not observed for Maternals. Overall, our findings demonstrate that producers should manage the nutrition of triplet-bearing Merino ewes so that ewes are in greater BCS at lambing and/or to gain BCS between pregnancy scanning and lambing to improve ewe and lamb survival. Triplet-bearing Maternal ewes should be managed to gain BCS between pregnancy scanning and lambing to improve ewe survival. Further research is needed to determine the optimal BCS profile for triplet-bearing ewes and the relationship between ewe BCS, time of death and cause of death. The impacts of over-conditioning triplet-bearing ewes on ewe and lamb survival should also be investigated, particularly for Maternals. The novel findings from this study will inform best-practice guidelines for the management of triplet-bearing ewes to improve ewe and lamb survival and animal welfare.

## Figures and Tables

**Table 1:** Total ewes, number of research sites and mobs, average (range) mob size (*n* ewes/mob) and stocking rate (ewes/ha) of ewes at lambing, and availability of shelter within the lambing paddocks (%) for triplet-bearing ewes of Maternal and Merino breed managed in the High and Low body condition score (BCS) treatments at 19 commercial research sites across southern Australia between 2019 and 2021.

Breed	BCS Treatment	Total Ewes	*n* Sites	*n* Mobs	Mob Size	Stocking Rate	Shelter ^1^
Maternal	High	1135	12	28	42	4.1	16
					(21–98)	(2.5–7.5)	(0–60)
	Low	1019		23	43	4.4	13
					(21–99)	(2.9–8.3)	(0–75)
Merino	High	545	7	22	25	3.5	18
					(11–50)	(0.1–9.5)	(0–50)
	Low	551		22	25	3.5	17
					(12–51)	(0.1–9.1)	(1–50)

^1^ Visual assessment of the proportion of the lambing paddock area occupied by shelter.

**Table 2:** Mean feed-on-offer (FOO; kg DM/ha) and proportion of legume in the pasture (%) pre-lambing and at marking for Maternal and Merino ewes in the High and Low body condition score (BCS) treatments at 19 commercial research sites across southern Australia between 2019 and 2021.

	Timepoint ^1^	Maternal		Merino	
		High BCS	Low BCS	High BCS	Low BCS
FOO	Pre-lambing	1541	1492	1412	1423
	Marking	1798	1813	1875	1784
Legume	Pre-lambing	29.4	30.1	36.6	33.1
	Marking	33.2	29.9	36.0	36.7

^1^ Pre-lambing assessments were made at, on average, 137 days from the start of joining and marking assessments were made at, on average, 165 days from the end of joining.

**Table 3:** Mean change in body condition score (BCS) between pregnancy scanning and pre-lambing and between pre-lambing and marking for triplet-bearing Maternal and Merino ewes managed at High and Low BCSs at 19 commercial research sites across southern Australia between 2019 and 2021. On average, measurements for pregnancy scanning were conducted at 97 days from the start of joining, pre-lambing measurements were conducted at 136 days from the start of joining, and marking measurements were conducted at 165 days from the end of joining. The average length of joining was 36 days.

	Maternal		Merino		l.s.d. between Breeds	l.s.d. within Breeds	*p*-Value ^1^
	High BCS	Low BCS	High BCS	Low BCS			
Scanning to pre-lambing	0.26	−0.25	0.01	−0.43	0.29	0.06	0.094
Pre-lambing to marking	−0.44	−0.07	−0.34	−0.06	0.24	0.09	0.134

^1^ *p*-value is for the interaction between breed and BCS treatment.

**Table 4:** Mean mortality (%) of triplet-bearing ewes and survival (%) of their lambs to marking for Maternal and Merino ewes managed at High and Low body condition scores (BCS) between pregnancy scanning and marking at 19 commercial research sites across southern Australia between 2019 and 2021. Data for ewe mortality were angular transformed and back-transformed values are presented in brackets.

	High BCS	Low BCS	l.s.d. ^1^ between Breeds	l.s.d. ^1^ within Breeds	*p*-Value ^1^
Ewe mortality					
Maternal	14.3 (6.1%)	12.7 (4.8%)	5.1	4.7	<0.01
Merino	11.8 (4.2%)	19.7 (11.4%)			
Lamb survival					
Maternal	56.2	59.3	7.2	4.1	<0.001
Merino	53.4	47.1			

^1^ *p*-values are for the interaction between BCS treatment and breed; the least significant differences for ewe mortality apply to the transformed data.

## Data Availability

The datasets generated and/or analysed during the current study are not publicly available but are available from the corresponding author on reasonable request pending permission from the funding body (Meat and Livestock Australia) and Murdoch University.
